# The Influence of Selected Laser Engraving Parameters on Surface Conditions of Hybrid Metal Matrix Composites

**DOI:** 10.3390/ma16196575

**Published:** 2023-10-06

**Authors:** Michał Szymański, Damian Przestacki, Paweł Szymański

**Affiliations:** 1 Faculty of Mechanical Engineering, Institute of Material Technology, Poznan University of Technology, ul. Piotrowo 3, 61-138 Poznan, Poland; michal.s.szymanski@put.poznan.pl (M.S.); pawel.szymanski@put.poznan.pl (P.S.); 2 Faculty of Mechanical Engineering, Institute of Mechanical Technology, Poznan University of Technology, ul. Piotrowo 3, 61-138 Poznan, Poland

**Keywords:** laser engraving, hybrid metal matrix composites, surface measurement, topography analysis, laser parameters

## Abstract

Hybrid metal matrix composites (HMMCs) are a special type of material, possessing combined properties that belong to alloys and metals according to market demands. Therefore, they are used in different areas of industry and the properties of this type of material are useful in engineering applications, e.g., in aircraft engines and electrotechnical parts. The structure of the material requires a number of scientific studies to develop an appropriate processing technology. The paper presents the susceptibility of material from the HMMCs group with the EN AC-44300 (AISi12(Fe)) aluminum alloy matrix with a two-component reinforcement made of alumina particles (AP) and aluminosilicate fibers (AF) to thermal treatment with a laser beam. During this process, laser engraving of the researched material with variable beam power *P_av_* and variable speed of the laser head *v_l_* were carried out. A metallographic analysis of the material was carried out. After laser engraving, surface structural changes of the material were determined. The properties of the surface geometric structure of processed material were also examined. Presented studies concern laser engraving on the surface of composite from the HMMC group, which was made by vacuum infiltration. Thanks to this method, it is possible both to produce shaped and precise composite castings with saturated reinforcement and to consequently minimize machining losses. Metal–ceramic composites from the HMMC group are hard-to-machine materials which create problems during machining. The aim of these studies was to develop a laser engraving technology with Al matrix composite with the addition of Al_2_O_3_ particles and aluminosilicate fibers, which constitute the reinforcement. The focus was on the selection of engraving parameters (beam power and speed of movement of the laser head). Clear examples of engraving, suitable for macro-assessment, were obtained with minimal change in the initial surface structure of the composite.

## 1. Introduction

The development of laser technology was not only concerned with welding or cutting operations but also with various surface modification technologies [1]. Various materials are processed with a laser beam [2,3]. Engraving consists of making recesses in the material of various depths to give the surface a changed permanent structure [4]. Nowadays, modern engraving methods are used such as etching with chemical substances, or the most modern of them, which is laser engraving [2,3]. Laser engraving is a method that is increasingly used for the industrial marking of elements and machine parts [5,6,7]. The quality of laser engraving was largely dependent on the roughness of the resulting surface [8]. Different values are expected depending on the application. For this reason, it is important to select the appropriate technological parameters of the process, such as beam power and speed of the laser head speed [9]. Examples of laser engraving studies on various materials have been described in the literature [6,7]. Qi et al. analyzed the laser marking process of stainless steel, studying the effect of laser pulse frequency on the depth and width of the beam pattern on the material [7]. An interesting study was carried out by Nikolidakis et al. [10] to develop a model used to predict the actual dimensions of engraved geometry, taking into account structural defects. The optimization of laser beam speed was tested by Wang et al. [11]. The glass marking process was carried out in the research conducted by Kuo et al. [12]. In turn, Noor and others subjected ceramics and plastics to laser engraving [13]. Genna et al., based on the previously prepared theoretical model of the process, subjected PMMA material to laser engraving, indicating the positive effect of increasing the speed of the laser head on the surface roughness of the processed material [14]. Zhou et al. demonstrated the increase in the depth of remelting with increasing power of the laser beam in the example of copper sheet processing. This method can be applied for the production of hydrogen microreactor elements [15]. Wu et al. studied the effect of technological parameters of the laser marking process, such as beam power, on the structure of the remelted polycrystalline diamond surface. These studies are important for the production of PCD tools [16]. Laser treatment is used for working materials that are difficult to machine by other methods [17,18]. As indicated, tests for surface treatment of different materials with laser beams are popular. However, few items in the available literature deal with the engraving of metal–ceramic composites. Zhang et al. conducted studies on the effect of laser beam action on the surface structure of Al warp composite reinforced with SiCp particles [19]. An Al alloy reinforced with graphene particles, which has been subjected to surface laser processing, has been studied by Prashantha et al. [20]. The influence of laser marking process parameters on the structure of Al–Si composite was studied by Rahimi et al. [21]. The tests [22] showed the possibility of improving the bond strengths of the composite materials. All analyzed test results related to composites produced by conventional casting methods, e.g., stir casting or pressure infiltration [23,24]. In the available literature, no mention has been found of successful attempts to mark metal composites with saturated reinforcement in vacuum infiltration condition [25].

Hybrid metal matrix composites are an advanced group of composite materials that are reinforced with two or more types of reinforcement [17]. HMMC materials are successfully used in the field of mechanical engineering [18], e.g., by aircraft engine parts [19] or overhead conductors [20]. One of the most popular composites in this group are materials based on an aluminum alloy matrix [21,26]. Due to their properties (low density, durability, and excellent dimensional stability), they are widely used in the engineering industry, such as the aviation, automotive, and building industries [18,19]. Such applications are conditioned by the plasticity of the matrix phase with the strength and hardness of the reinforcement phase [24,27]. Ceramic particles are characterized by high specific strength, damping properties, and high wear resistance [28].

Laser engraving is a very popular method of marking various parts in mechanical engineering. Considering the possibility of using the HMMC material in the production of machine parts, it is reasonable to develop a laser engraving technology for this material. The melting point of alumina ceramics is approx. 2000 °C and for AlSi12 it is about 660 °C. Due to such large differences in melting point values, the impact of laser engraving on the structure of the HMMC material should be determined.

The studies carried out concern laser engraving on the surface of HMMC composite by vacuum infiltration [29,30,31], which involves the saturation of porous ceramic reinforcement with liquid metal under vacuum conditions. The ceramic reinforcement already at the shaping stage has a pre-finished target shape of the finished composite casting, which makes it possible to produce precise and shaped castings [31]. Thanks to this, the finishing machining is reduced to a minimum [32,33]. In addition, this method can be used to manufacture machine parts, e.g., shafts and gears, whereas in the available literature, there is no information on the manufacture of such parts during testing. Other researchers limited themselves to casting small fittings or produced castings using methods much more expensive in terms of production technology [18,34].

Many scientists are studying various aspects of the laser engraving of different types of metal alloys. However, none of the previous works have attempted to study the technological parameters of the laser beam on hybrid metal matrix composites with saturated reinforcement during laser engraving. It can be added that laser engraving tests are of great cognitive importance to this manufacturing process in order to fully apply the composite as a structural material. The main purpose of the presented research was to analyze the impact of technological parameters of laser engraving, such as the power and speed of the laser head, on surface roughness. During the tests, a material from the hybrid metal matrix composites group made of AlSi12/AP/AF was used. Both the selection of components of the tested composite and their quantitative ratio were preceded by earlier studies by the authors.

The marking of individual parts of machinery, in practice, is the only method of distinguishing individual parts of machinery from each other. Laser marking of HMMC composites is an important process for the industry due to the problematic recycling of metal–ceramic composites [35,36]. It is impossible to distinguish between aluminum alloy and aluminum–matrix composite parts even with spectrographic analysis due to the presence of silicon atoms in the reinforcement. Additionally, metal–ceramic composites require different recycling procedures.

## 2. Materials and Methods

### 2.1. Hybrid Metal Matrix Composite and EN AW 1050A Aluminum Alloy (Technical Aluminum)

The presented research focuses on the examination of the laser engraving process of a metal matrix composite made of AP (FEPA 100) with the addition of AF saturated under vacuum with the AlSi12 alloy and technical aluminum for comparison. The microstructure analysis of HMMC material was carried out on the NIKON Eclipse MA200 metallographic microscope (Tokyo, Japan).

**Figure 1:**
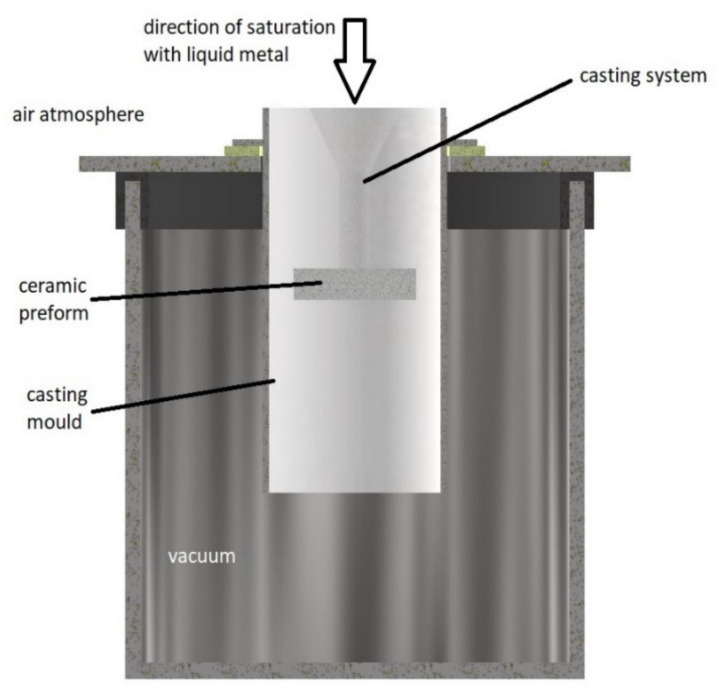
Scheme of the process of vacuum infiltration (it contains a ceramic fitting with a pouring system in a gypsum form placed in a vacuum chamber).

The tested composite material was produced in the process of vacuum infiltration (Figure 1). The vacuum infiltration method is carried out by saturating a previously prepared ceramic preform with liquid metal, which is deposited in a gypsum mold located in a steel sleeve.

**Figure 2:**
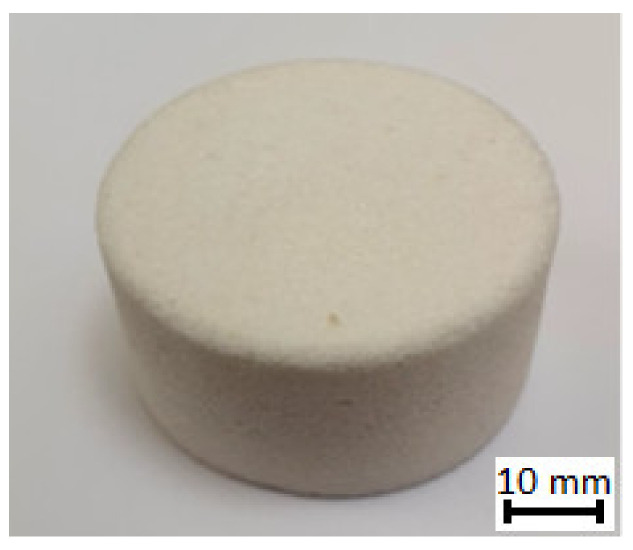
Porous ceramic preform with Al_2_O_3_ mass.

The ceramic preform is shaped using a 3D printed mold made in the rapid prototyping process. By using 3D printing to produce printed molds, it is possible to give the final shape of the casting at the stage of manufacturing the porous ceramic preform. The procedure for preparing ceramics in printed matrices was preceded by earlier studies [31,33]. The first step involves firing polystyrene with a mass of Al_2_O_3_ and SiO_2_ in a mold-firing furnace, which is equipped with a heated outflow for molten polymers, special filters, and ventilation. The second step involves annealing ceramics in a high-temperature ceramic furnace. Figure 2 shows ceramic preform with Al_2_O_3_ mass prior to the firing process to illustrate the process. After annealing at 1000 and complete curing, the ceramic was impregnated with paraffin and poured with casting gypsum mass.

The casting mold prepared in this way is placed in a vacuum chamber in which a negative pressure is generated. In this way, air is removed from the porous ceramic preform. The next step is to introduce the liquid metal alloy through the gating system into the preform. The saturation of the reinforcing phase takes place in the process of equalizing the pressure in the vacuum chamber with the atmospheric pressure. The pressure difference between the vacuum chamber and the atmosphere created in this way causes the saturation of the porous preform with the liquid matrix.

**Figure 3:**
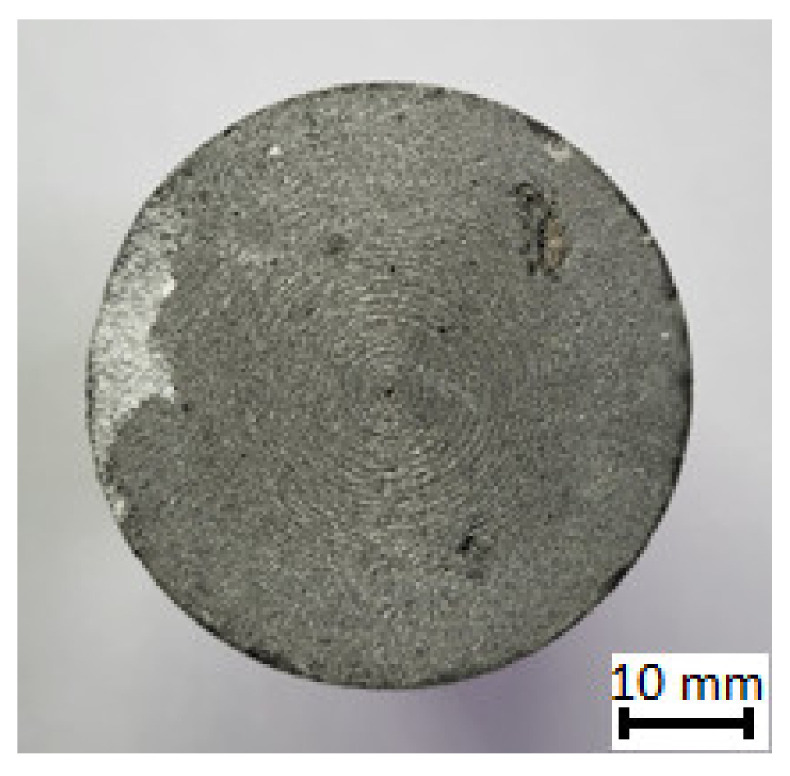
Initially prepared for laser engraving composite casting.

An important advantage of such a process is the ability to effectively saturate the reinforcement without additional external pressure. By using 3D printing to produce printed molds, it is possible to give the final shape of the casting at the stage of manufacturing the porous ceramic preform. As a result, it is possible to obtain composite precision castings with a hybrid reinforcement phase of complicated shapes. Moreover, this casting method is not expensive. This gives a significant advantage over the use of other methods. The finished casting (Figure 3), whose geometry was designed using CAD software, reflects the shape given when forming the ceramic preform in a 3D printed mold.

**Figure 4:**
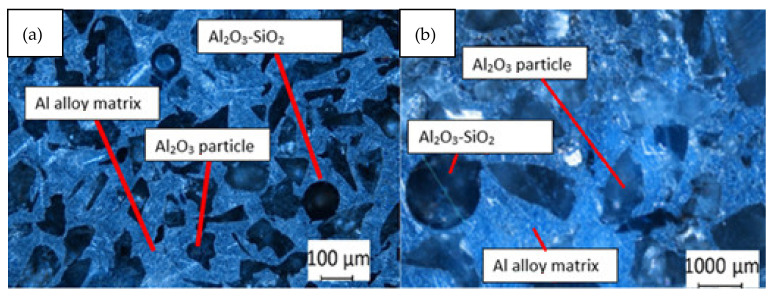
Metallographic structure made with optical microscope of hybrid metal matrix composite made of AP (FEPA 100) with the addition of AF saturated under vacuum with AlSi12 alloy (**a**) at a smaller magnification and (**b**) at a larger magnification.

Aluminum alloy AlSi12 consists of 12.3% Si, 0.7% Fe, 0.5% Mn, 0.15% Zn, 0.15% Ti, and 0.1% Cu. The ceramic reinforcement consists of the following components: 97% of the loose mass of the mixture is ordinary aluminum oxide with a grain size of 125 to 130 [μm] (FEPA 100) and a hardness of 9 Mohs scale. The second component of the reinforcement constituting 3% of the mass is aluminosilicate fiber of the 1260 grade, ø10 [μm], and hardness 6 on the Mohs scale. Sodium water glass was added as a preliminary binder during the manufacture. Ceramic reinforcement grains have an irregular shape (Figure 4). 

### 2.2. Laser Engraving and Measurement Processes

The laser engraving process was carried out on the MaxBox laser from TYKMA Electrox (Chillicothe, OH, USA). It is a device built on the basis of Yb:Fiber technology so it is a fiber laser doped with ytterbium. It emits a laser beam with a wavelength of 1060–1080 nm. The pulse power of the beam emitted by this device reaches 120 W.

The study of the geometric structure of the machined surface was carried out using the JENOPTIC HOMMEL-ETAMIC W5 profilometer (Jena, Germany).

**Figure 5:**
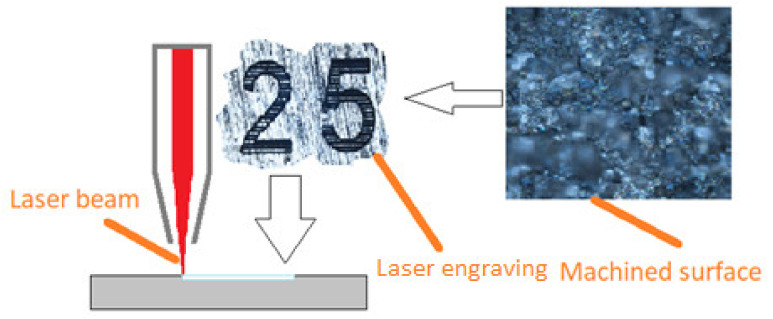
The diagram of the laser engraving process.

Image analysis of the surface structure was carried out using a NIKON (Tokyo, Japan) Eclipse MA200 metallographic microscope too. The diagram of the laser engraving process is shown in Figure 5.

A sample of both materials was prepared for testing in the form of a disc with a diameter of 50 mm and a thickness of 7 mm. The surface was ground. The laser engraving process was carried out with constant parameters, namely pulse frequency f = 12 kHz, laser beam focal diameter D = 50 μm, and pulse time ti = 220 ns. The variable parameters were the pulse power of the laser beam *P_i_* and the speed of the laser head *v_l_*. The table below (Table 1) contains the individual processing parameters of the HMMCs and technical aluminum.

The geometric structure of the surface of each sample was tested five times. The arithmetic average deviation of the profile from the mean line *Ra* and the height of the roughness according to ten points of the *Rz* profile were examined. Measurements were made on a measuring section of l = 4.8 mm.

## 3. Results and Discussion

### 3.1. Surface Structure Optical Measurement

**Figure 6:**
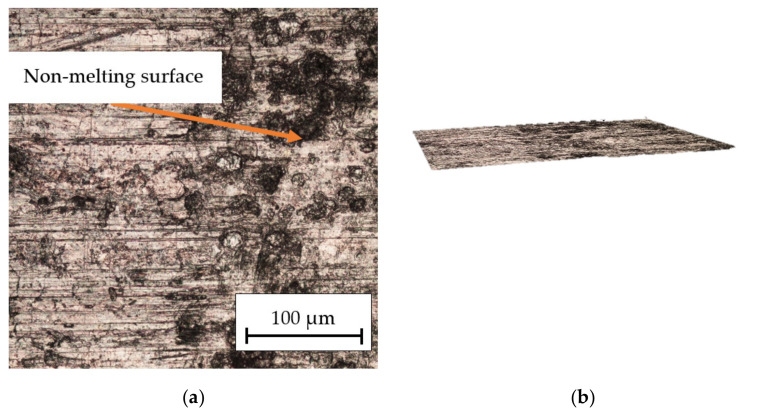

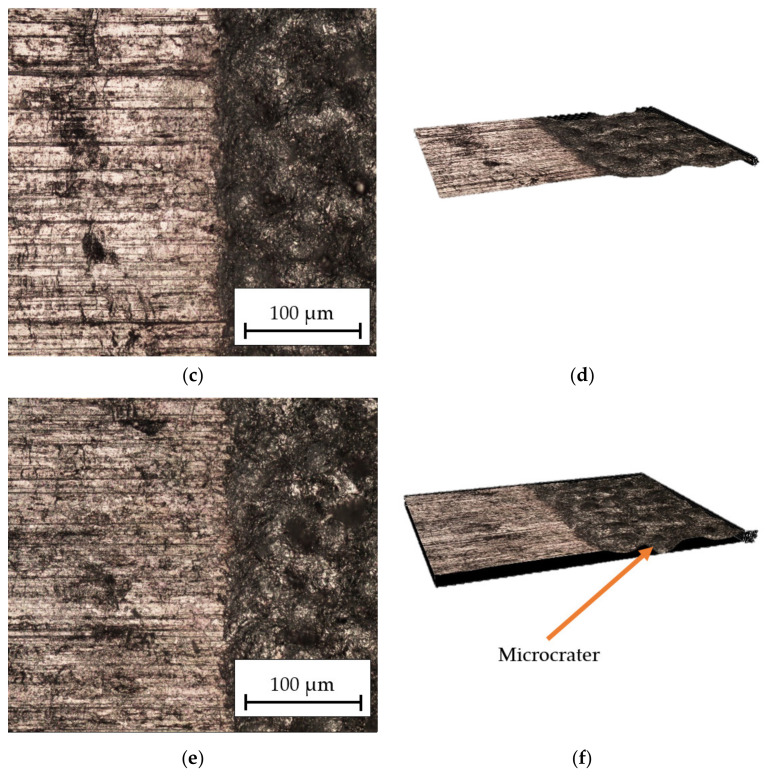
Optical microscope picture of the surface structure of aluminum alloy at 30× and 100× magnification: (**a**,**b**) *P_av_* = 15.84 W, *v_l_* = 50 mm/s; (**c**,**d**) *P_av_* = 23.76 W, *v_l_* = 50 mm/s; and (**e**,**f**) *P_av_* = 31.68 W, *v_l_* = 50 mm/s.

The results analysis in Figure 6 indicates the influence of the laser beam power on the surface structure of the technical aluminum. It was found that for the power of *P_av_* = 15.84 W (Figure 6a,b), only slight traces of the laser beam’s passage through the material were obtained. In some places, there was no melting of the surface. A regular structure with visible wide grooves after the passage of the laser beam was observed after increasing the power to *P_av_* = 23.76 W (Figure 6c,d). Figure 6e,f shows the machined surface after an increase in laser power to *P_av_* = 31.68 W. Significant melting of the material, especially at the intersection of the grooves after the laser beam, could be observed. The microcrater (Figure 6f) is probably the result of a casting defect in the form of porosity occurring in this place. The effect is due to the process parameters (melt and mold temperature, injection and pressure, and injection speed) that influence the microstructure of the material.

**Figure 7:**
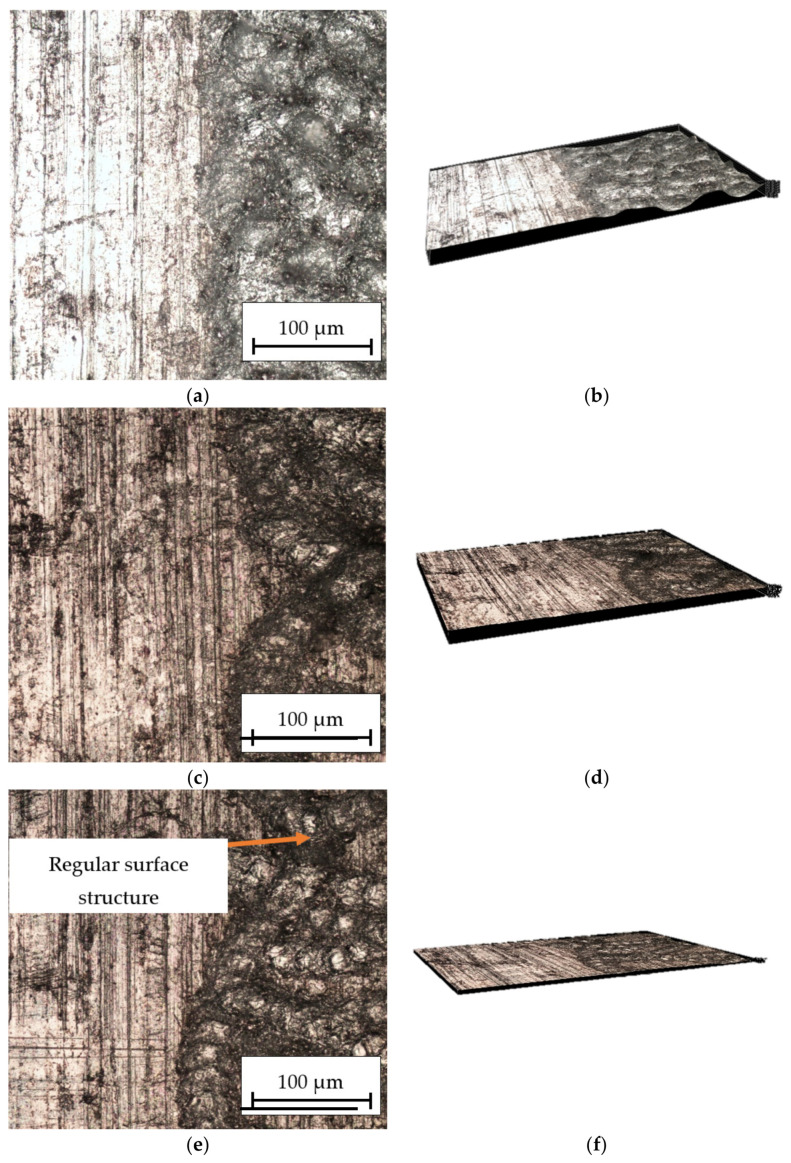
Optical microscope picture of the surface structure of aluminum alloy at 30× and 100× magnification: (**a**,**b**) *P_av_* = 23.76 W, *v_l_* = 50 mm/s; (**c**,**d**) *P_av_* = 23.76 W, *v_l_* = 150 mm/s; and (**e**,**f**) *P_av_* = 23.76 W, *v_l_* = 250 mm/s.

Figure 7 shows the influence of the speed of the laser beam on the surface structure of the technical aluminum. After machining with *v_l_* = 50 mm/s (Figure 6a,b), the very fine structure with a significant degree of melting of the material surface could be observed because the grooves were near together and wide. In the case of the speed *v_l_* = 150 mm/s (Figure 7c,d), the distance between the grooves was increased. A lower degree of layer melting was observed. Increasing the engraving speed to *v_l_* = 250 mm/s (Figure 7e,f) led to an increase in the distance between the grooves. Due to the shorter time of heating of the surface with a laser beam, the passages are narrow and there are no depressions in the material structure.

**Figure 8:**
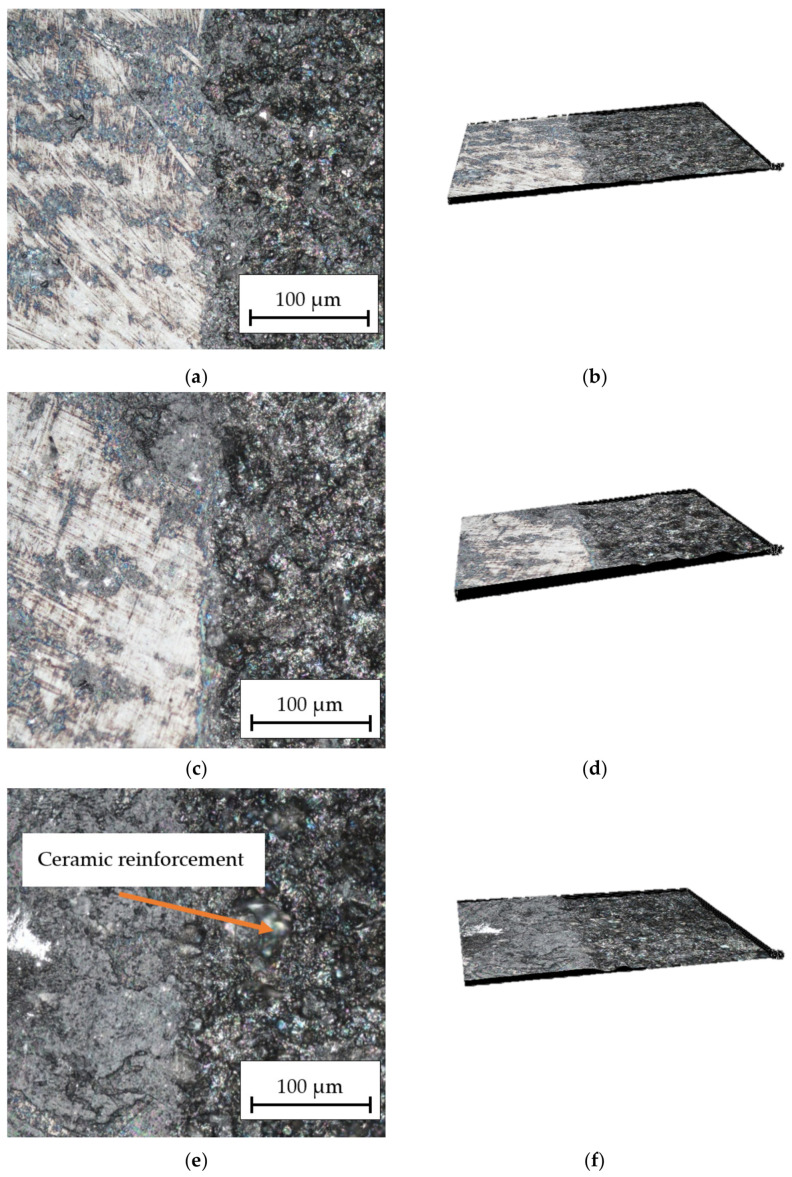
Optical microscope picture of the surface structure at 30× and 100× magnification for the HMMCs: (**a**,**b**) *P_av_* = 15.84 W, *v_l_* = 50 mm/s; (**c**,**d**) *P_av_* = 23.76 W, *v_l_* = 50 mm/s; and (**e**,**f**) *P_av_* = 31,68 W, *v_l_* = 50 mm/s.

The obtained results in Figure 8 show the influence of the laser beam power on the surface structure of the HMMCs with the AlSi12 matrix. With increasing power, the material was etched more and more and only a small part of it was removed. After machining HMMCs with *P_av_* = 15.84 W, a small melting of the surface layer was observed. Increasing the laser beam power to *P_av_* = 23.76 W results in greater penetration on the material surface. There are no depressions in the path traveled by the laser beam in the form of grooves on any of the samples, although in Figure 8e,f you can see a very delicate outline of the beam travel. Ceramic reinforcement in the form of bright spots is visible in Figure 8e.

**Figure 9:**
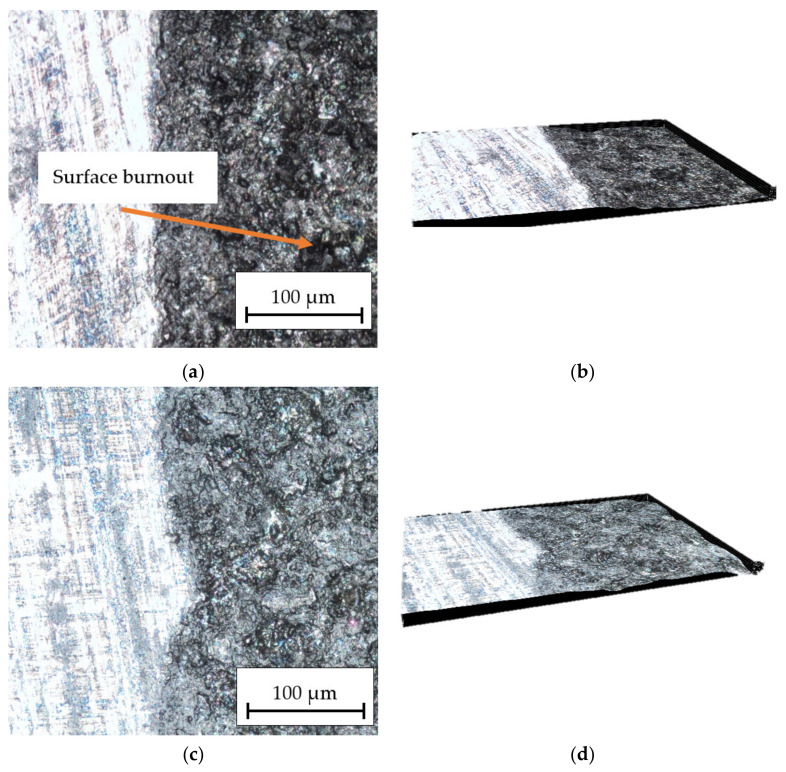

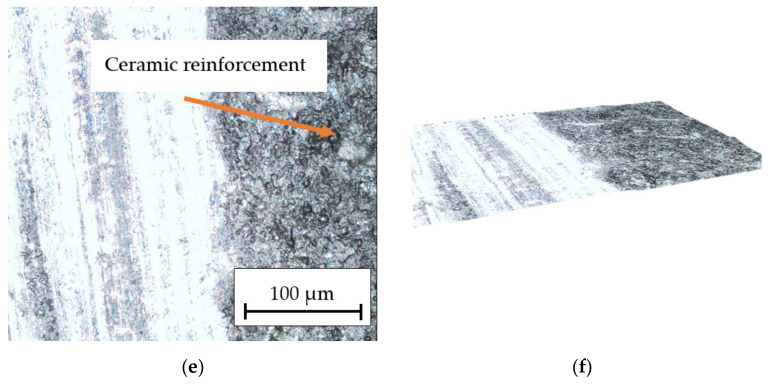
Optical microscope picture of the surface structure at 30× and 100× magnification for the HMMC composite: (**a**,**b**) *P_av_* = 23.76 W, *v_l_* = 50 mm/s; (**c**,**d**) *P_av_* = 23.76 W, *v_l_* = 150 mm/s; and (**e**,**f**) *P_av_* = 23.76 W, *v_l_* = 250 mm/s.

The obtained results in Figure 9 show photos of the HMMCs composite with AlSi12 matrix after engraving at different speeds. At the speed of *v_l_* = 50 mm/s (Figure 9a,b), a small outline of the path of the laser beam is visible and the material is etched over the entire machined surface. When the beam speed *v_l_* = 150 mm/s (Figure 9c,d) is used, the etched material is slightly less well etched and unetched material is visible in some places. Increasing the speed to *v_l_* = 250 mm/s (Figure 9e,f) resulted in a much weaker etching of the material and an increase in the distance between successive passes of the laser beam. In addition, surface burnout (Figure 9a), caused by the local concentration of laser beam energy and elements of ceramic reinforcement in the form of bright spots, is visible in Figure 9e. Places of uneven absorption of the laser beam energy are visible, which is caused by the heterogeneous structure of the material.

After the tests of laser engraving of the composite material from the HMMCs group and aluminum alloy, the study of the geometric structure of the surface of the processed material was carried out.

### 3.2. Geometrical Structure of the Surface after Laser Engraving of HMMC

**Figure 10:**
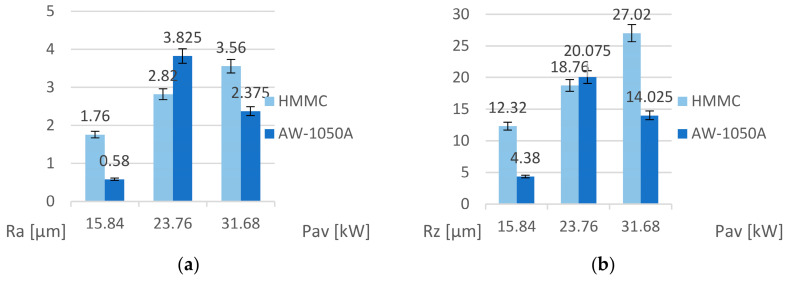
Graph of dependence of roughness parameters on average beam power *P_av_*: (**a**) *Ra* parameter and (**b**) dla *Rz* parameter.

The starting point for further analysis is the results of measurements of Ra and Rz surface parameters of the samples. The obtained results in Figure 10 present the values of the *Ra* and *Rz* roughness parameters depending on the average beam power *P_av_*. For the HMMCs, it can be determined that the roughness parameters increase proportionally to the increase in the average power of the laser beam due to the presumably increasing amount of thermal energy accumulated by the ceramic grains. In the case of the aluminum alloy, the lowest surface roughness was obtained after marking with a beam of power *P_av_* = 15.84 W and the highest when marking with a beam of power *P_av_* = 23.76 W. Only in the case of the intermediate value of the laser beam power was the alloy roughness higher than that of the hybrid metal matrix composites.

**Figure 11:**
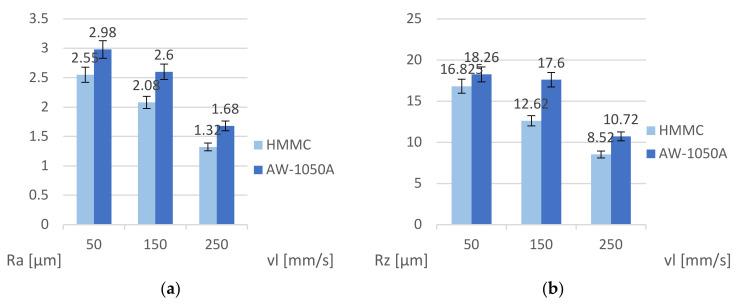
Graph of dependence of roughness parameters on speed of the laser head *v_l_*: (**a**) *Ra* parameter and (**b**) *Rz* parameter.

The results analysis in Figure 11 indicates a correlation of the *Ra* and *Rz* roughness parameters with the machining speed *v_l_*. These parameters for the HMMCs and AW-1050A decrease with increasing speed *v_l_.* During each measurement, higher roughness was obtained on aluminum samples. Comparative analysis of the geometric structure of the surface of the tested materials based on variable process parameters allowed us to select the optimal conditions for laser engraving of a new material, which is a composite from the HMMCs group.

### 3.3. Examples of Laser Engraving of HMMC

**Figure 12:**
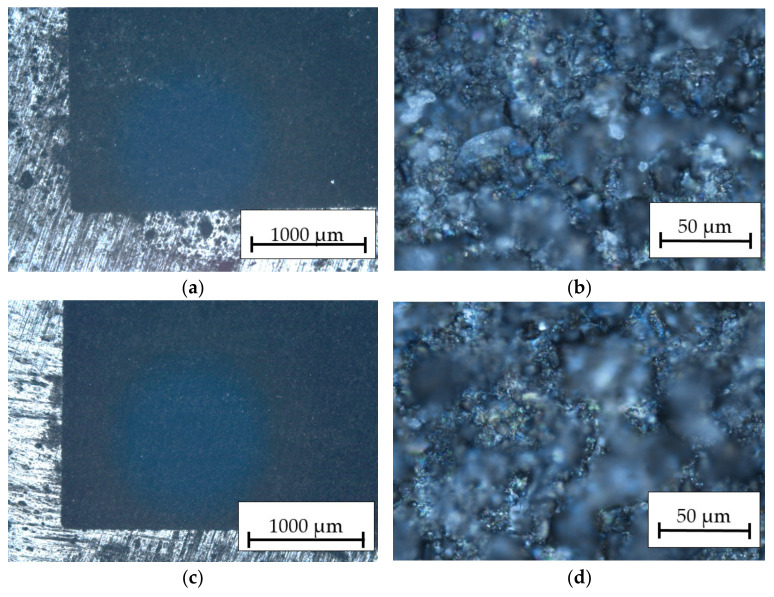

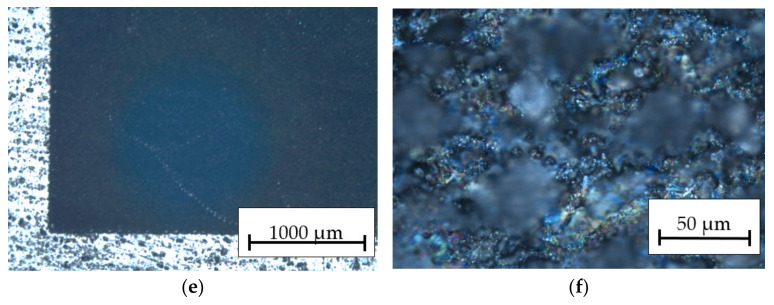
Optical microscope picture of the surface structure at 30× and 100× magnification for the HMMCs: (**a**,**b**) *P_av_* = 15.84 W, *v_l_* = 50 mm/s; (**c**,**d**) *P_av_* = 23.76 W, *v_l_* = 50 mm/s; and (**e**,**f**) *P_av_* = 31.68 W, *v_l_* = 50 mm/s.

**Figure 13:**
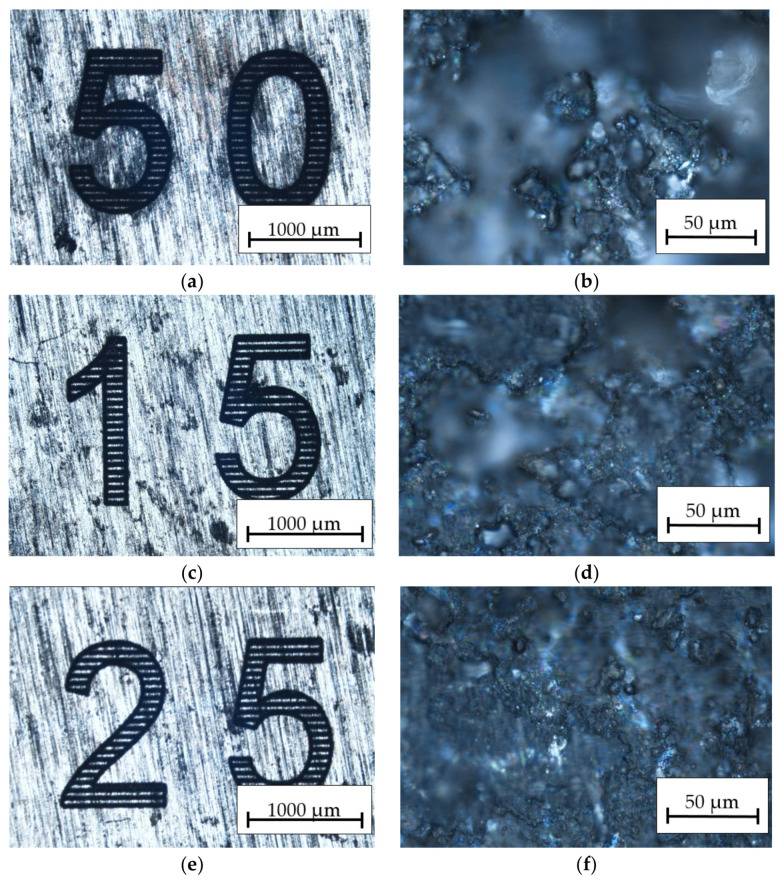
Optical microscope picture of the surface structure at 30× and 100× magnification for the HMMCs: (**a**,**b**) *P_av_* = 23.76 W, *v_l_* = 50 mm/s; (**c**,**d**) *P_av_* = 23.76 W, *v_l_* = 150 mm/s; and (**e**,**f**) *P_av_* = 23.76 W, *v_l_* = 250 mm/s.

After researching the laser engraving technology of the HMMCs and selecting the optimal process conditions for the aluminum alloy, it was decided to carry out marking tests by applying specific markings to the surface of the material. The results analysis in Figure 12 indicates the attempts to mark the HMMCs with variable beam power, while Figure 13 shows the attempts to mark the composite with variable speed of the laser head.

As the laser power increases, the roughness of the machined surface of the HMMCs with the AlSi12 alloy matrix increases. This is the result of an increasing degree of surface etching. The increase in processing speed during laser engraving affects the trajectory of the laser beam, increasing the distance between successive parallel passes. Increasing the processing speed reduces the pulse density. The effect of this phenomenon is to shorten the exposure time of the material to the laser beam. For the HMMCs with the AlSi12 alloy matrix, the increase in the processing speed leads to a decrease in the values of the Ra and Rz parameters. The tests also showed that in the case of marking with a beam power of *P_av_* = 23.76 W, it is possible to obtain a lower surface roughness of the HMMCs than in the case of the aluminum alloy. Increasing the speed of the beam at this power, despite the lower melting of the material surface, reduces the surface roughness.

## 4. Conclusions

The analysis of the results allow the following conclusions to be formulated.

- The tests showed that the hybrid metal matrix composites with the AlSi12 alloy matrix is difficult to laser process because the heterogeneous structure makes it difficult to select technological parameters of the process due to different absorption of thermal energy by the matrix and reinforcement components;
- The best engraving effects were obtained at *v_l_* = 250 mm/s and *P_av_* = 23.76 W;
- Changes in the surface structure of the metallic matrix and ceramic grains of the HMMCs are visible;
- The research was aimed at determining whether and to what extent it would be possible to obtain a clear engraving on the surface of the HMMCs. Due to the heterogeneity of the HMMCs material structure, full fusion is not achieved with lower energy of the laser beam. With appropriate beam power and higher speed of the laser head, a less melted surface is obtained and thus, a surface with less roughness;
- Engraving of HMMC composite materials AlSi12/AP/AF requires the selection of parameters allowing for minimal interference in the structure of the material surface as a result of the supplied thermal energy;
- As a result of the studies carried out, examples of laser engraving of HMMC composite with minimal impact on its structure were obtained, which are suitable for macro-assessment.

Laser engraving changes the structure of the surface layer of the composite cast in the marking area. This process may also change the structure of the matrix material and thus influence its operational properties, such as resistance to abrasive wear or corrosion, as well as mechanical properties.

It is necessary to conduct research in this direction which will be the aim of the authors’ investigations in future works.

## Figures and Tables

**Table 1:** Variable parameters during laser engraving of the HMMCs and technical aluminum.

Test No.	Laser Beam Pulse Power *P_i_*	Speed of the Laser Head *v_l_*
[-]	[W]	[mm/s]
1.	6	50
2.	9	50
3.	12	50
4.	9	50
5.	9	150
6.	9	250

## Data Availability

Not applicable.

## Nomenclature

HMMC	hybrid metal matrix composite
AF	alumina fibers
AP	alumina particles
PCD	polycrystalline diamond
*P_av_*	power of laser beam (W)
*v_l_*	speed of the laser head (mm/s)
Ra	arithmetic mean deviation from the mean line (μm)
Rz	highest profile height (μm)
